# Supplementary material for: Robust detection of oncometabolic aberrations by 1H–13C heteronuclear single quantum correlation in intact biological specimens
Source: Commun Biol. 2020 Jun 25;3:328. doi: 10.1038/s42003-020-1055-5 (PMC7316726; doi:10.1038/s42003-020-1055-5)
Supplement: Supplementary file 1 — Supplementary Information [file 42003_2020_1055_MOESM1_ESM.pdf]

Supplementary Information

Title: Robust detection of oncometabolic aberrations by ^{1}H-^{13}C heteronuclear single quantum correlation in intact biological specimens

Supplementary Figure 1

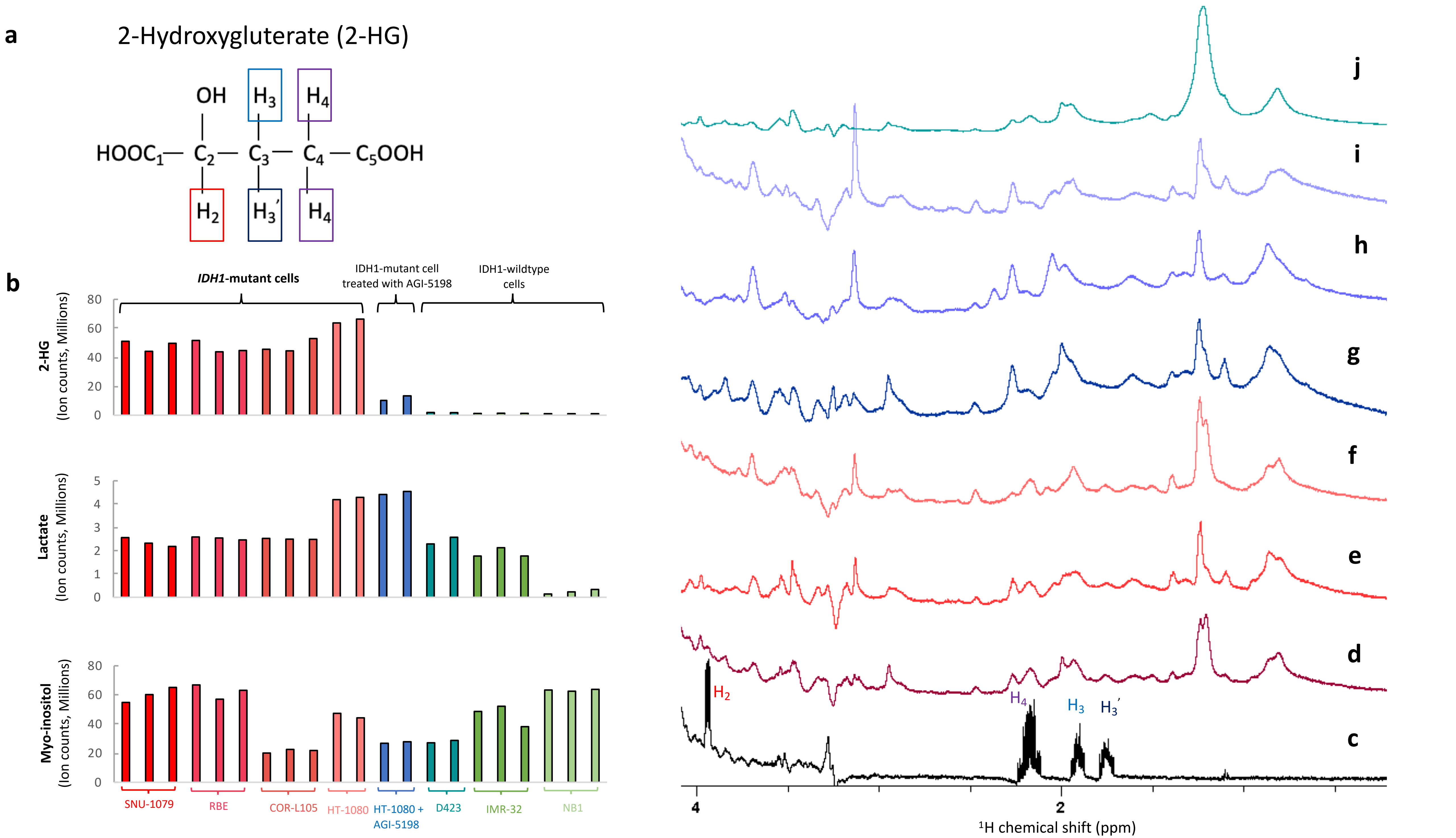

Supplementary Figure 1: 2-HG is dramatically elevated in mutant *IDH1* cells but the detection of 2-HG specific peaks is unreliable in the ^{1}H spectrum due to extensively convolution by signals from high abundant metabolites. (**a**) The molecular structure of 2-HG. (**b**) The mass spectroscopy data comparing the level of 2-HG, lactate and Myo-inositol in *IDH1* mutant cells (SNU-1079, RBE, CORL-105 and HT-1080), HT1080 cells treated with 10uM mutant IDH1 inhibitor (AGI-5198) and wildtype cells (D423, IMR32 and NB1). The average value of 2-HG in *IDH1* mutant cells is (51+/-7) *10^6 ion counts while this value for HT-1080 cell treated with inhibitor is (11+/-1.6)*10^6 ion counts and for *IDH1* wildtype cells is (1+/-0.5)*10^6 ion counts. While the level of lactate and myo-inositol do not vary significantly between *IDH1* and wildtype cells. Based on mass-spect data, 2-HG is high abundant metabolite in *IDH1* mutant cells therefore it can be detected by magnetic resonance spectroscopy. ^{1}H spectrum of (**c**) 2-HG standard and its peaks assignment, (**d**) live R132C *IDH1* mutant cells (HT-1080), (**e**) live R132H *IDH1* mutant cells (NHA *IDH1*), (**f**) live R132C *IDH1* mutant cells (SNU1079), (**g**) live R132C *IDH1* mutant cells (HT-1080) treated with 10uM mutant IDH1 inhibitor (AGI-5198) for 48hrs (**h**) live R132H *IDH1* mutant cells (NHA m*IDH1*) treated with 10uM mutant IDH1 inhibitor (AGI-5198) for 48hrs (**i**) live R132C *IDH1* mutant cells (SNU-1079) treated with 10uM mutant IDH1 inhibitor (AGI-5198) for 48hrs (**j**) live *IDH1* wild type cells (D423). Due to the peaks broadening and presence of other highly abundant metabolites, ^{1}H spectrum does not allow reliable detection of 2-HG peaks.

Supplementary Figure 2

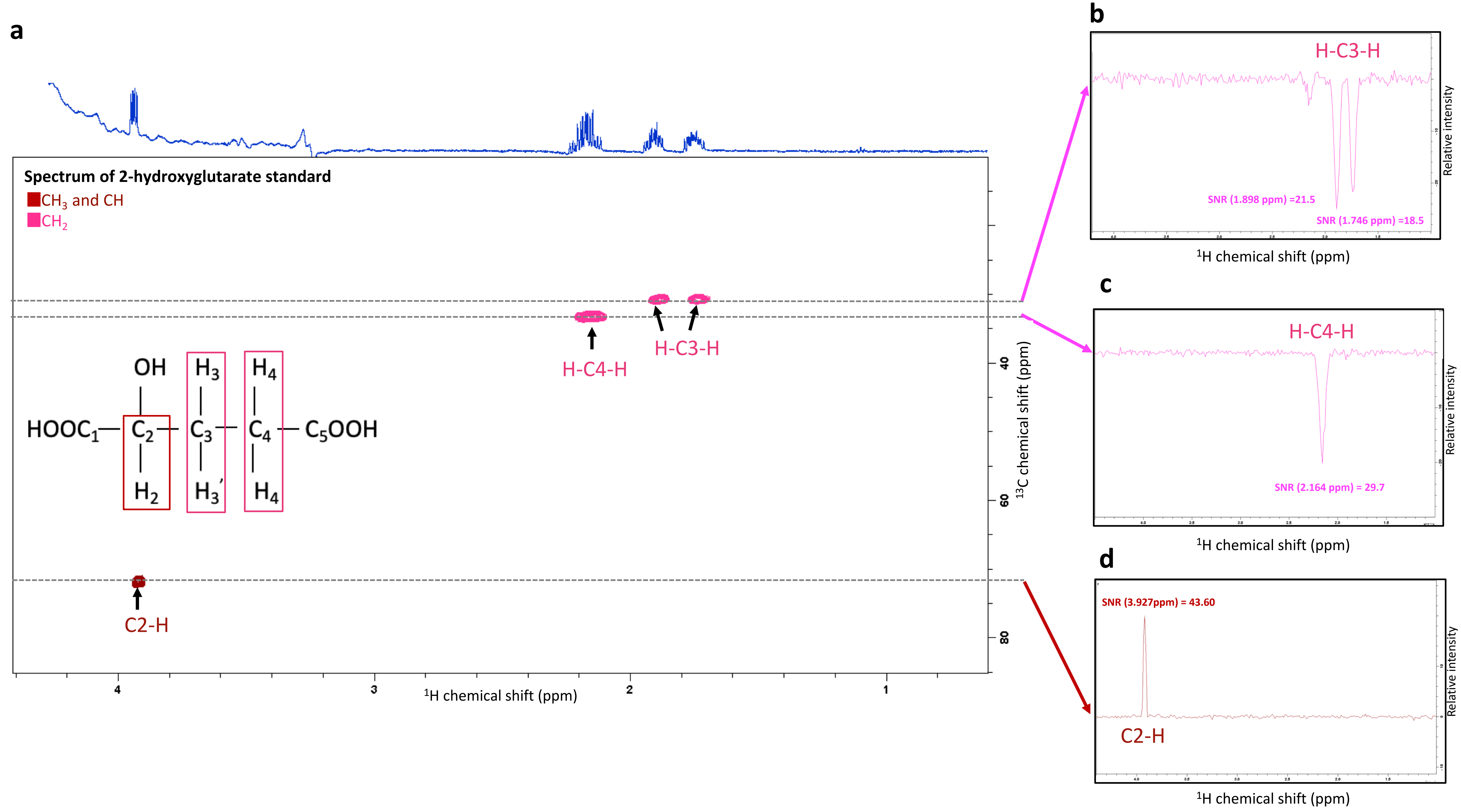

Supplementary Figure 2: Signal to noise ratio (SNR) analysis of specific rows obtained from the HSQC spectrum of individual peaks in 2-HG chemical standard. Phase sensitive ^{1}H-^{13}C HSQC spectrum of a chemical standard 2-HG at 7.5mM concentration in 90% PBS and 10% D_2O using the HSQCEDETGPSISP2.3 pulse sequence, where the first color code in each spectrum depicts peaks which have positive phase (CH_3 and CH) and the second color code shows peaks which have negative phase (CH_2). The spectrum is acquired using 500 MHz Bruker AVANCE III NMR. The 1D ^{1}H spectrum of 2-HG standard displayed as the x-axis on top of the 2D HSQC spectrum. In ^{1}H-^{13}C HSQC, the protons which are directly bounded with ^{13}C atoms are detected, these carbons and protons are labeled in the 2-HG structure, and peaks are also annotated in the spectrum. The C2-H peak in the phase sensitive ^{1}H-^{13}C HSQC spectrum has the positive phase, while the other peaks have negative phase (H-C-H). Phases of each peak can be more appreciated from their specific row containing each peak as shown on panels b-d. The signal to noise ratio for each peak is acquired by adjusting the phase of negative peaks to positive and selecting the region of interest and applying SINO command in Topspin. The small bump in the spectrum of S2b is caused by the impurity in our standard.

Supplementary Figure 3

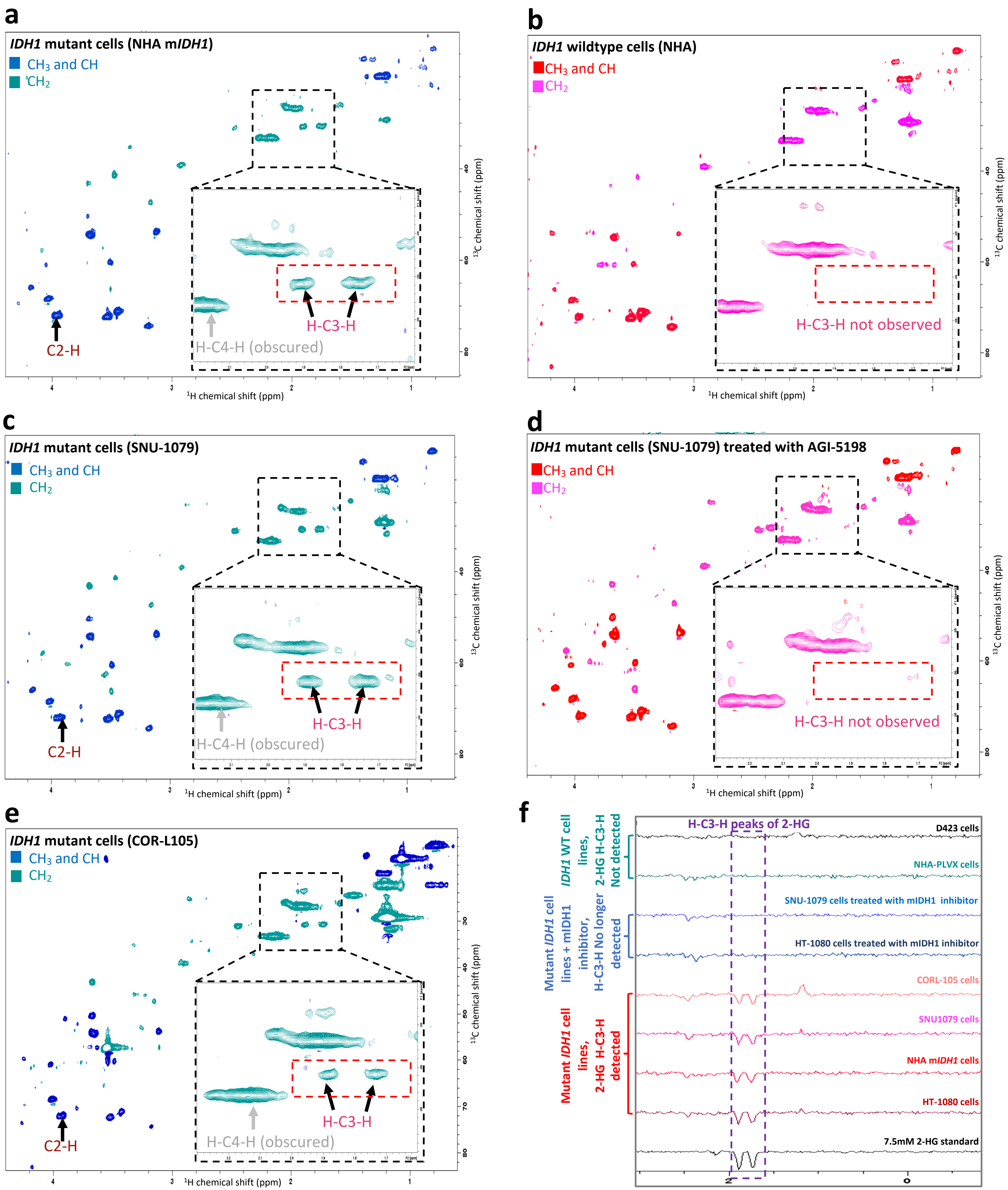

Supplementary Figure 3: H-C3-H peaks of 2-HG are uniquely detected in ^{1}H-^{13}C HSQC spectra of *IDH1*-mutant cells and are eliminated by mutant IDH1 specific inhibitor treatment. Phase sensitive ^{1}H-^{13}C HSQC spectra using HSQCEDETGPSISP2.3 pulse sequence, where the first color code in each spectrum depicts the peaks which have positive phase (CH_3 and CH) and the second color code shows the peaks which have negative phase (CH_2), acquired using 500 MHz Bruker AVANCE III NMR. The red dashed box shows where we expect to see H-C3-H peaks of 2-HG in the spectrum. (**a**) The spectrum of live *IDH1* mutant cells (immortalized normal human astrocyte, NHA m*IDH1*) cells which express mutant *IDH1*. (**b**) The ^{1}H-^{13}C HSQC spectrum of live immortalized normal human astrocyte cells (NHA) which express non-mutant *IDH1*. (**c**) The ^{1}H-^{13}C HSQC spectrum of *IDH1* mutant cells (SNU-1079). (**d**) The ^{1}H-^{13}C HSQC spectrum of *IDH1* mutant cells (SNU-1079) treated with 10uM mutant IDH1 inhibitor (AGI-5198) for 48hrs. (**e**) The spectrum of live *IDH1* mutant cells (CORL-105). The presence of 2-HG peaks is readily evident in spectra of all *IDH1* mutant cells (**a**,**c** and **e**)**.** C2-H peak of 2-HG has the chemical shift close to the highly abundant myo-inositol peak, and barely distinguishable. The H-C4-H peak of 2-HG is obscured by the broad (-CH2-) lipid peak. However, H-C3-H peaks of 2-HG are easily detectable in *IDH1* mutant cells spectra. The spectrum of live *IDH1* wildtype cells (NHA) shows complete absence of H-C3-H peaks of 2-HG (**b**)**.** Also, treating cells with mutant IDH1 inhibitor results in disappearance of H-C3-H peaks of 2-HG (**d**)**.** (**f**) Shows the specific row of H-C3-H peaks of 2-HG extracted from ^{1}H-^{13}C HSQC spectrum of 2-HG standard, live mutant *IDH1* cells, live mutant *IDH1* cells treated with mutant IDH1 inhibitor and live *IDH1* wildtype cells. The specific rows extracted from 2D HSQC spectrum of mutant *IDH1* cells show the negatively phased doublet associated with H-C3-H peaks of 2-HG. The spectra are normalized to the noise level.

Supplementary Figure 4

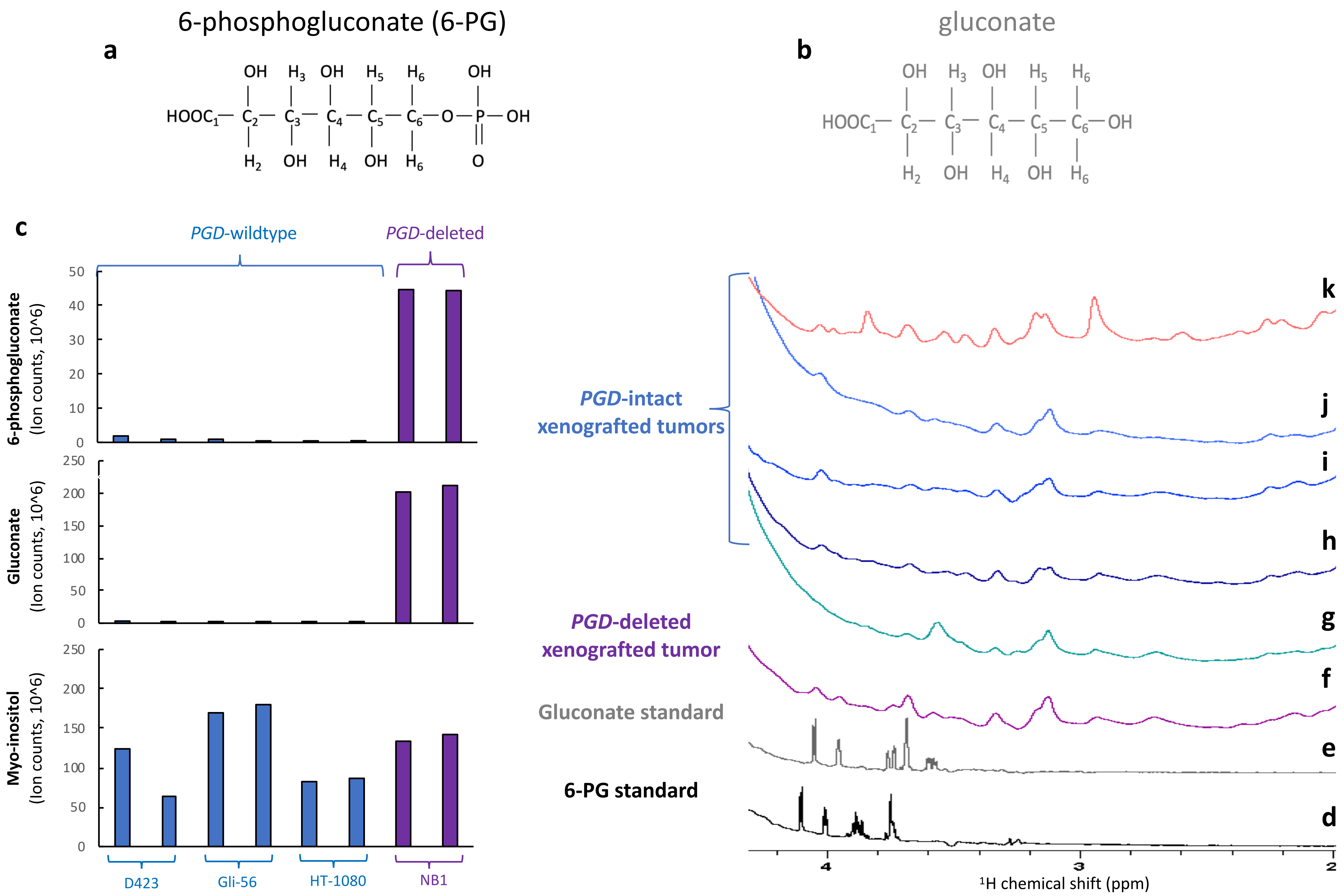

Supplementary Figure 4: Gluconate and 6-phosphogluconate are highly abundant metabolites in *PGD*-deleted cells but are indistinct in the ^{1}H spectrum of *PGD*-deleted tumors because of nearness of the water signal and spectral convolution by high abundant metabolites: (**a**) The molecular structures of gluconate and (**b**) 6-phosphogluconate (6-PG). (**c**) mass-spec data comparing the levels of 6-phosphogluconate, gluconate and lactate in extracts of *PGD*-deleted vs wildtype cells indicates that *PGD*-deletion leads to a dramatic accumulation of 6-PG/gluconate. Yet, 6-PG or gluconate are not readily identifiable and do not distinguish the ^{1}H spectra of *PGD*-deleted xenografted tumors *ex-vivo* from those that are *PGD*-intact or rescued. ^{1}H spectrum of (**d**) 9 mM 6-PG standard and (**e**) 9 mM gluconate standard in 90% PBS and 10% D_2O (**f**) *PGD*-deleted tumor (NB1) and (**g**) *PGD*-rescued tumor (NB1-*PGD*) (**h**) *PGD*-wildtype tumors (D423) (**i**) *PGD*-wildtype tumor (G59) (**j**) *PGD*-wildtype tumor (U87) (**k**) normal mouse brain. Inability to visualized 6-PG/gluconate in *PGD*-deleted tumors is most likely due to spectral convolution by chemical shifts from other highly abundant metabolites and closeness of gluconate chemical shifts to the water signal.

Supplementary Figure 5

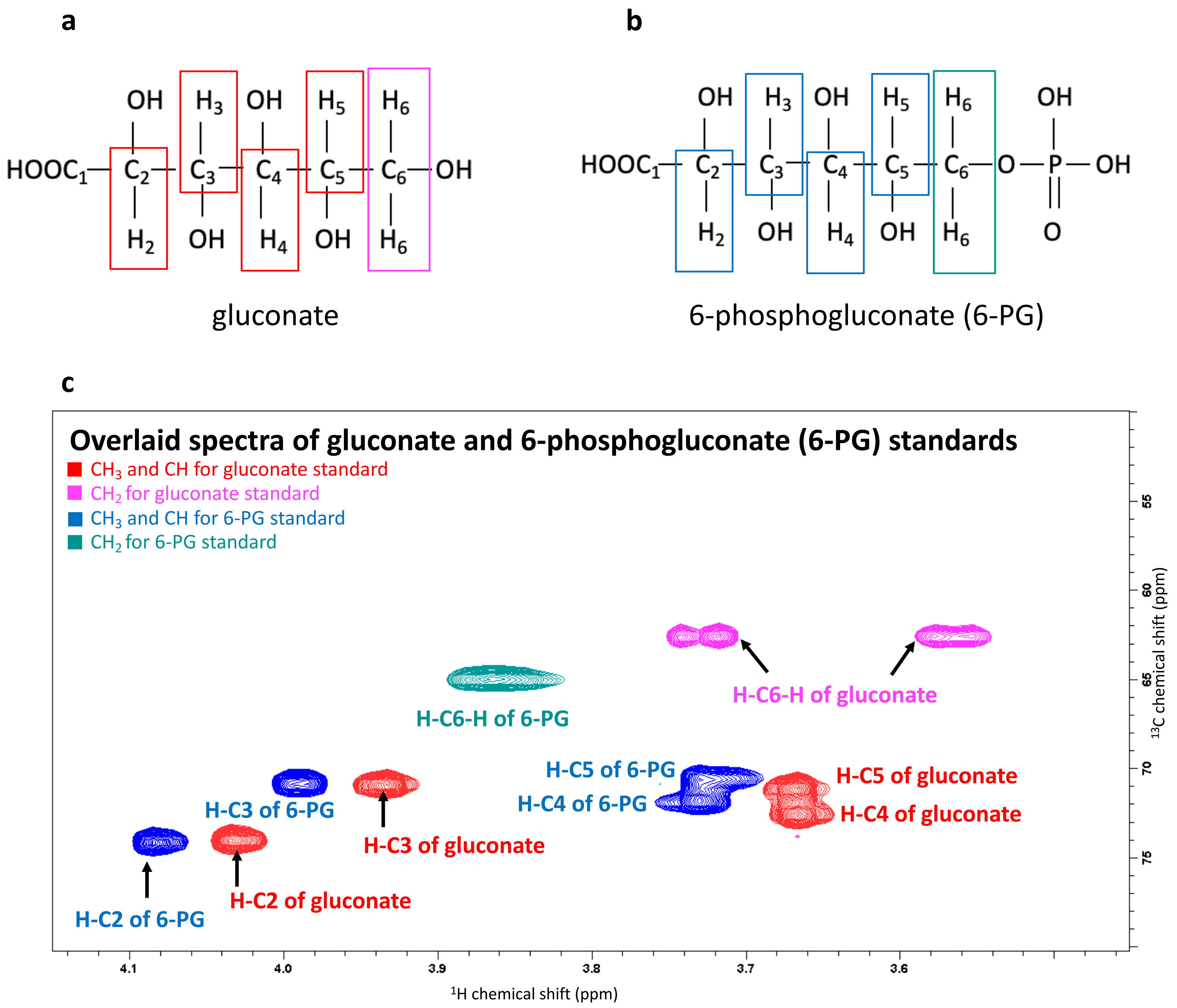

Supplementary Figure 5: Phase sensitive ^{1}H-^{13}C HSQC spectrum of gluconate and 6-phosphogluconate standard. (**a**) Molecular structure of gluconate. (**b)** Molecular structure of 6-phosphogluconate (6-PG). (**c**) The overlaid phase sensitive ^{1}H-^{13}C HSQC spectra of 9 mM gluconate and 6-PG standard in 90% PBS and 10% D_2O using HSQCEDETGPSISP2.3 pulse sequence. Red and blue peaks are associated with CH_3 and CH groups of gluconate and 6-PG, respectively and have positive phase while purple and green peaks are associated with CH_2 group of gluconate and 6-PG, respectively and have negative phase. Human metabolomics data base (HMBD) was consulted for gluconate peaks assignment[1-4].

Supplementary Figure 6

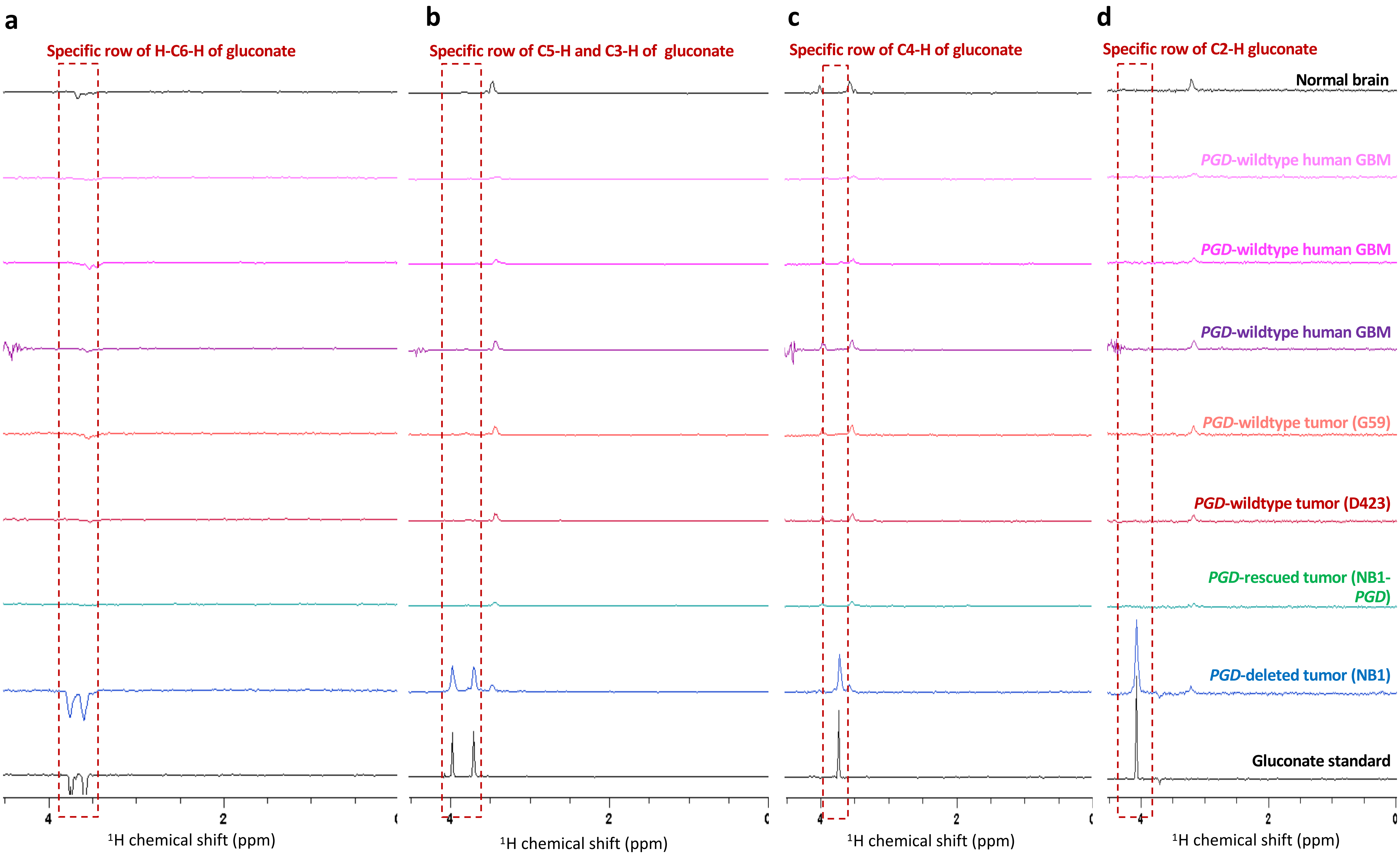

Supplementary Figure 6: Specific detection of gluconate in *PGD*-deleted but not *PGD*-rescued or WT tumors. The specific rows of (**a**) H-C6-H peaks (**b**) C5-H and C3-H (**c**) C4-H and (**d**) C2-H of gluconate from the phased sensitive ^{1}H-^{13}C HSQC spectrum of 9 mM gluconate standard, *PGD*-deleted tumors, *PGD*-rescued tumors, *PGD* wildtype tumors (xenografted and human GBM) and normal mouse brain. Specific rows extracted from 2D HSQC spectrum of *PGD*-deleted tumors show the peaks associated with gluconate peaks. While these peaks are absent in *PGD*-rescued and wildtype tumors and normal mouse brain. The spectra are normalized to the noise level.

Supplementary Figure 7

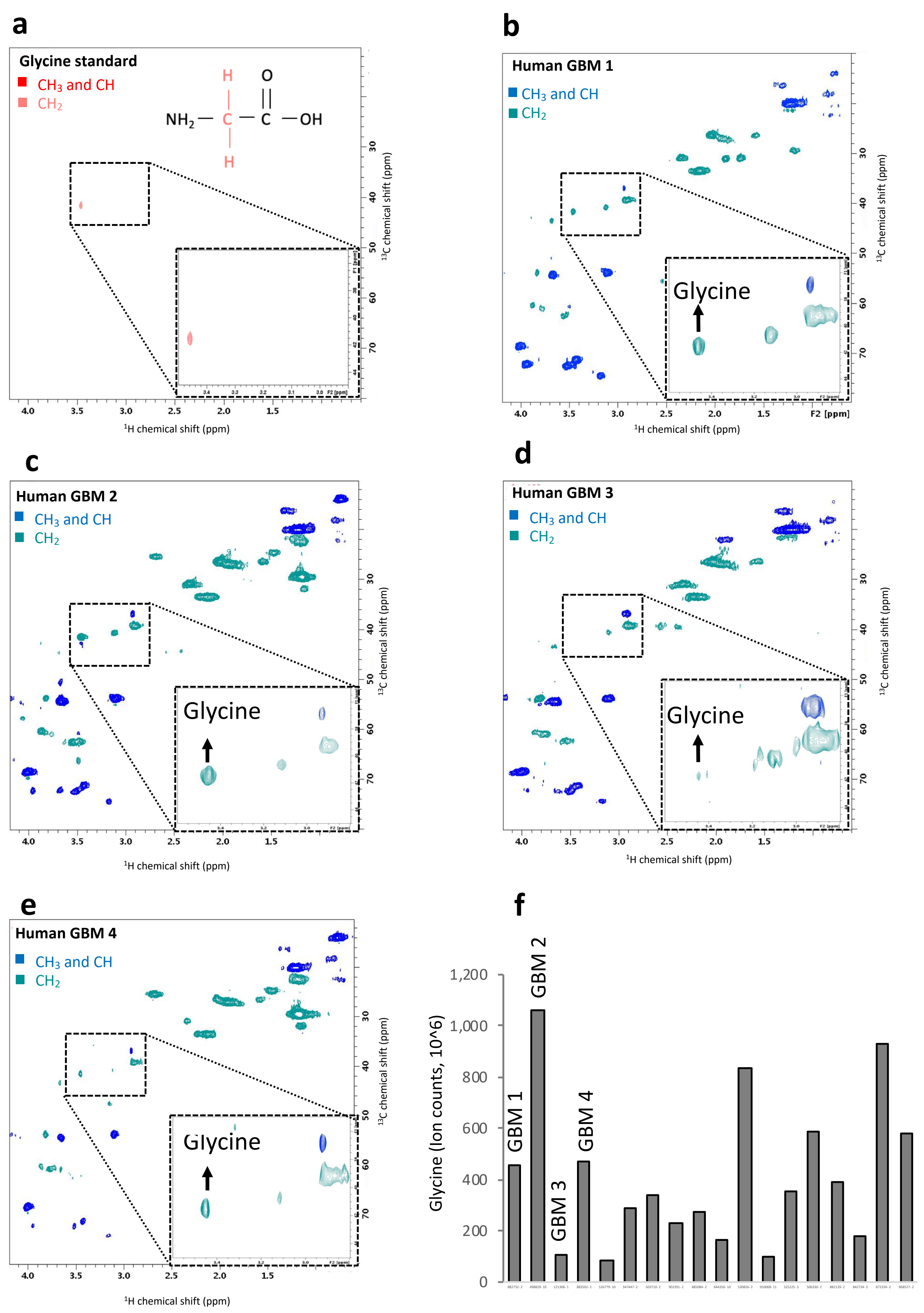

Supplementary Figure 7: Glycine, a metabolite associated with poor prognosis, can be readily detected in intact human GBMs spectra despite high abundance metabolites with similar shifts. Phase sensitive ^{1}H-^{13}C HSQC spectra of human GBMs using HSQCEDETGPSISP2.3 pulse sequence, where the first color code in each spectrum depicts the peaks which have positive phase (CH_3 and CH) and the second color code shows the peaks which have negative phase (CH_2), acquired using 500 MHz Bruker AVANCE III NMR. (**a**) spectrum of glycine standard in 90% PBS and 10% D_2O. The peak at (3.45 ppm, 41.5 ppm) corresponds to the only carbon bounded hydrogen atom of glycine. (**b**-**e**) Spectra of human GBMs. (**f**) The mass spectroscopy data from metabolon Inc. comparing the level of glycine in human GBMs. Glycine peak in the spectrum of GBM3 is barely above the detection threshold. The glycine peak in GBM2 is stronger than other GBM's spectra which is consistent with mass spectrometry data.

Supplementary Figure 8

Cancer Cells remain viable for the time course of the NMR HSQC scan

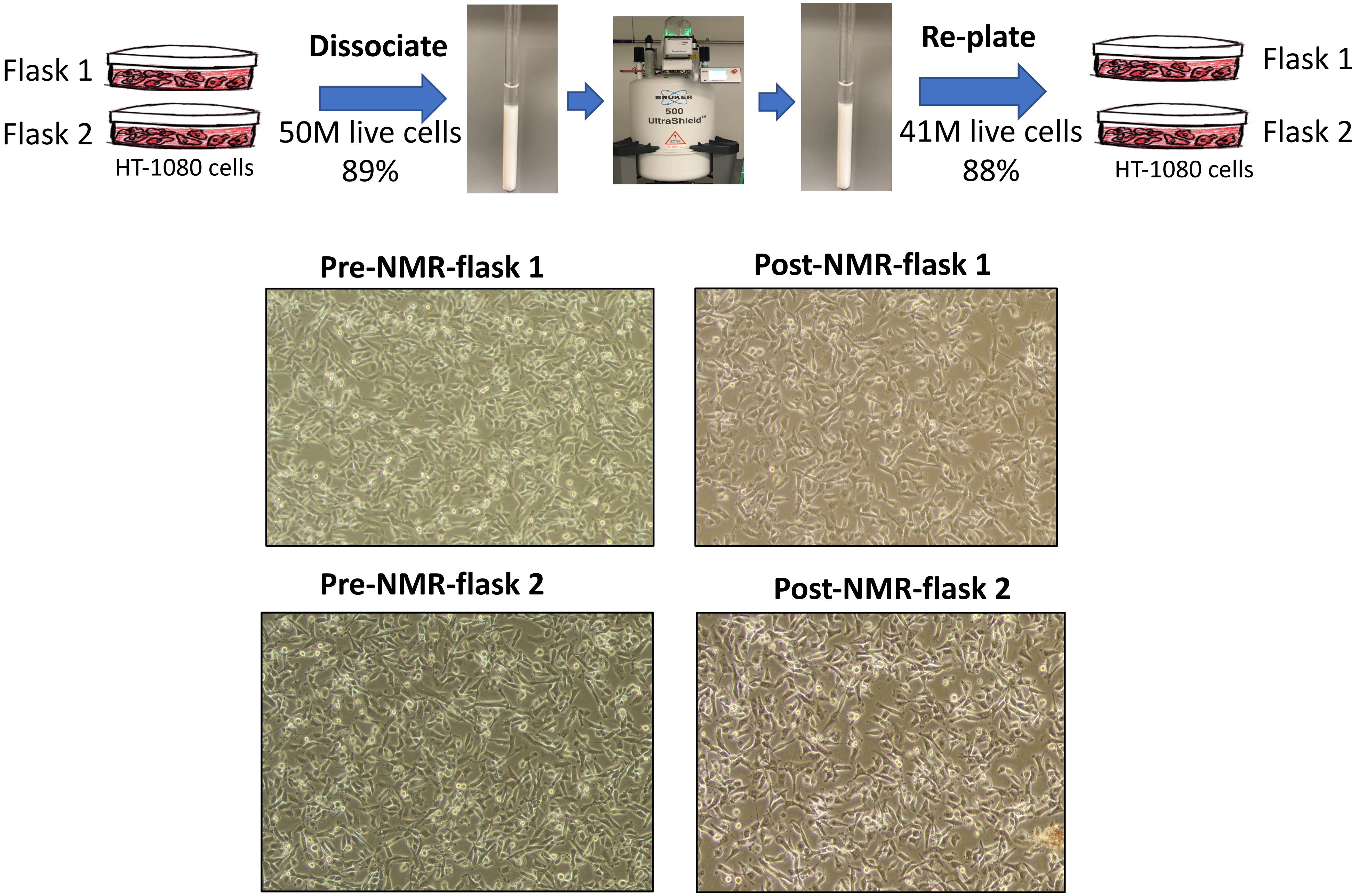

Supplementary Figure 8: Cancer Cells remain viable for the time course of the NMR HSQC scan. (a) Cells were cultured in 15 cm plates. At 90% confluency, cells were harvested and washed once with 1x PBS to remove residual media. Viable cell number was measured using Trypan Blue. A suspension of 170 µL cells (40-50 million cells) was then re-suspended in 500 µL of a solution containing 90% PBS with 10% D_2O with 3% TSP and transferred to 5 mm NMR tubes. NMR tubes were sealed with parafilm. The spectral acquisition began immediately after sample preparation. Once measurements were complete, the viable cell number was measured again using Trypan Blue to verify cell viability during the duration of the experiment. (**b**) Additionally, to verify cell plating efficacy after NMR, cells were re-plated on 15 cm culture plates (photomicrographs shown).

References

1 Wishart, D. S. *et al.* HMDB: the Human Metabolome Database. *Nucleic Acids Res* **35**, D521-526, doi:10.1093/nar/gkl923 (2007).

2 Wishart, D. S. *et al.* HMDB: a knowledgebase for the human metabolome. *Nucleic Acids Res* **37**, D603-610, doi:10.1093/nar/gkn810 (2009).

3 Wishart, D. S. *et al.* HMDB 3.0--The Human Metabolome Database in 2013. *Nucleic Acids Res* **41**, D801-807, doi:10.1093/nar/gks1065 (2013).

4 Wishart, D. S. *et al.* HMDB 4.0: the human metabolome database for 2018. *Nucleic Acids Res* **46**, D608-d617, doi:10.1093/nar/gkx1089 (2018).
