# Supplementary material for: Robust detection of oncometabolic aberrations by 1H–13C heteronuclear single quantum correlation in intact biological specimens
Source: Commun Biol. 2020 Jun 25;3:328. doi: 10.1038/s42003-020-1055-5 (PMC7316726; doi:10.1038/s42003-020-1055-5)
Supplement: Supplementary file 2 — Description of Additional Supplementary Items [file 42003_2020_1055_MOESM2_ESM.pdf]

Description of Additional Supplementary Files

File Name: **Supplementary Data 1**
Description: Metabolomics source data for Figure 3e, Supplementary Figures S1b, S4c, and S7f
