# Supplementary material for: Robust detection of oncometabolic aberrations by 1H–13C heteronuclear single quantum correlation in intact biological specimens
Source: Commun Biol. 2020 Jun 25;3:328. doi: 10.1038/s42003-020-1055-5 (PMC7316726; doi:10.1038/s42003-020-1055-5)
Supplement: Supplementary file 4 — Reporting Summary [file 42003_2020_1055_MOESM4_ESM.pdf]

nature research

Corresponding author(s): Florian Muller

Last updated by author(s): 2020-29-4

Reporting Summary

Nature Research wishes to improve the reproducibility of the work that we publish. This form provides structure for consistency and transparency in reporting. For further information on Nature Research policies, see Authors & Referees and the Editorial Policy Checklist.

Statistics

For all statistical analyses, confirm that the following items are present in the figure legend, table legend, main text, or Methods section.

n/a	Confirmed	
☐	☒	The exact sample size (*n*) for each experimental group/condition, given as a discrete number and unit of measurement
☐	☒	A statement on whether measurements were taken from distinct samples or whether the same sample was measured repeatedly
☒	☐	The statistical test(s) used AND whether they are one- or two-sided<br>*Only common tests should be described solely by name; describe more complex techniques in the Methods section.*
☒	☐	A description of all covariates tested
☒	☐	A description of any assumptions or corrections, such as tests of normality and adjustment for multiple comparisons
☒	☐	A full description of the statistical parameters including central tendency (e.g. means) or other basic estimates (e.g. regression coefficient) AND variation (e.g. standard deviation) or associated estimates of uncertainty (e.g. confidence intervals)
☒	☐	For null hypothesis testing, the test statistic (e.g. *F*, *t*, *r*) with confidence intervals, effect sizes, degrees of freedom and *P* value noted<br>*Give P values as exact values whenever suitable.*
☒	☐	For Bayesian analysis, information on the choice of priors and Markov chain Monte Carlo settings
☒	☐	For hierarchical and complex designs, identification of the appropriate level for tests and full reporting of outcomes
☒	☐	Estimates of effect sizes (e.g. Cohen's *d*, Pearson's *r*), indicating how they were calculated

Our web collection on statistics for biologists contains articles on many of the points above.

Software and code

Policy information about availability of computer code

Data collection	NMR spectra were aquirred on a Brucker NMR and were analyzed with Bruker's Topsin software. This is a standard in the field and should prove no barrier for other researchers to review our data
Data analysis	NMR data analysis was performed with Topsin, which is both commercially available from Bruker (Berilica, MA).

For manuscripts utilizing custom algorithms or software that are central to the research but not yet described in published literature, software must be made available to editors/reviewers. We strongly encourage code deposition in a community repository (e.g. GitHub). See the Nature Research guidelines for submitting code & software for further information.

Data

Policy information about availability of data

All manuscripts must include a data availability statement. This statement should provide the following information, where applicable:

- Accession codes, unique identifiers, or web links for publicly available datasets
- A list of figures that have associated raw data
- A description of any restrictions on data availability

Source data for all figures are included as supplementary Data 1 (Metabolomics) and NMR data are available in the Figshare repository [https://figshare.com/articles/NMR_data_zip/12375203]. NMR data can be analyzed using NMR software such as TopSpin which can be downloaded for free from Bruker website. All other data are available from the corresponding author upon reasonable request.

Field-specific reporting

Please select the one below that is the best fit for your research. If you are not sure, read the appropriate sections before making your selection.

- [x] Life sciences
- [ ] Behavioural & social sciences
- [ ] Ecological, evolutionary & environmental sciences

For a reference copy of the document with all sections, see nature.com/documents/nr-reporting-summary-flat.pdf

Life sciences study design

All studies must disclose on these points even when the disclosure is negative.

Sample size	Sample size of the number of independently interrogated biological samples was limited by the number of cell lines with the desired genotype available.
Data exclusions	Except for gross technical mistakes (broken NMR tubes, spilled or unloaded samples) no data were excluded
Replication	Replication studies were performed both across genotypes and within genotypes (Summarized in Table I).
Randomization	No randomization was performed as this would not be practical
Blinding	No systematic and deliberate blinding of the experimentalist was performed; however, the experimentalist was not typically aware of what the genotype of a given sample was, thus

Reporting for specific materials, systems and methods

We require information from authors about some types of materials, experimental systems and methods used in many studies. Here, indicate whether each material, system or method listed is relevant to your study. If you are not sure if a list item applies to your research, read the appropriate section before selecting a response.

Materials & experimental systems

n/a	Involved in the study	
[x]	[ ]	Antibodies
[ ]	[x]	Eukaryotic cell lines
[x]	[ ]	Palaeontology
[ ]	[x]	Animals and other organisms
[ ]	[x]	Human research participants
[x]	[ ]	Clinical data

Methods

n/a	Involved in the study	
[x]	[ ]	ChIP-seq
[x]	[ ]	Flow cytometry
[x]	[ ]	MRI-based neuroimaging

Eukaryotic cell lines

Policy information about cell lines

Cell line source(s)	Cell lines were purchased from ATCC, DTP NCI-60 and MD Anderson's cell line core as well as obtained through MTA's from the original scientist (G-59)
Authentication	Cell lines were authenticated by STR testing at MD Anderson's Characterized Cell Line Core
Mycoplasma contamination	Cell lines were routinely tested for mycoplasma contamination using a commercial ELISA kit
Commonly misidentified lines (See ICLAC register)	LN319 was used as one of several IDH1-WT control cell lines; LN319 may be contaminated with LN992; Because LN992 is also IDH1-WT, this possible cross-contamination does not confound interpretation. U-87, obtained from ATCC, was used as another IDH1-WT control. Issues around mismatch between U87-ATCC and the original donor have been described; however, this is not an issue for interpretation, as U87-ATCC is confirmed as IDH1-WT. No other cell lines used in this study were found in the misdentified cell line list. Regardless, production of 2-HG was verified independtly for each cell line, regardless of what its proper name is.

Animals and other organisms

Policy information about studies involving animals; ARRIVE guidelines recommended for reporting animal research

Laboratory animals	Xenografted tumors in immunocompromised Foxn1 nude mice were employed between the ages of 7-25 weeks, female mice from M.D. Anderson's Department of Experimental Radiation Oncology (ERO).

Wild animals	NA
Field-collected samples	NA
Ethics oversight	All procedures were approved by M.D. Anderson's Institutional Care and Use Committee (IACUC)

Note that full information on the approval of the study protocol must also be provided in the manuscript.

Human research participants

Policy information about studies involving human research participants

Population characteristics	De-identified, archival GBM tumors were studied as part of the manuscript. As a result of de-identification, no demographic information is available.
Recruitment	GBM were consented and collected during resection surgeries under the approved institutional review board (IRB) protocol by the MD Anderson (PA15-0940; PI: De Groot).
Ethics oversight	M.D. Anderson's Institutional Review Board (PA15-0940)

Note that full information on the approval of the study protocol must also be provided in the manuscript.
